# Exploring the experiences of mental health professionals engaged in the adoption of mobile health technology in Irish mental health services

**DOI:** 10.1186/s12888-021-03426-5

**Published:** 2021-08-19

**Authors:** Ruth Melia, Luke Monahan, Jim Duggan, John Bogue, Mary O’Sullivan, Karen Young, Derek Chambers, Shane McInerney

**Affiliations:** 1 Health Service Executive, Dublin, Ireland; 2 National University of Ireland, Galway, Ireland; 3 Irish Management Institute, Dublin, Ireland

**Keywords:** Mobile health technology, mHealth, Mental health, Technology adoption

## Abstract

**Background:**

The World Health Organization report that an estimated 793,000 people died by suicide in 2016 globally. The use of digital technology has been found to be beneficial in the delivery of Web-based suicide prevention interventions. Research on the integration of digital technology within mental health services has indicated that despite the proliferation of technology, engagement by patients and professionals in adopting such technology can be poor.

**Objectives:**

The current study aims to explore the experiences of 15 mental health professionals involved in integrating mobile health technology into their practice. A secondary aim was to identify the drivers and barriers to the adoption of such technology by mental health professionals, and to consider what theoretical models could best account for the data.

**Methods:**

Semi-structured interviews, conducted from July to October 2019, were used to explore the experiences of mental health professionals engaged in the adoption of mobile health technology within mental health services. Mental Health professionals and clinician managers working in HSE Child and Adolescent Mental Health, Adult Mental Health, and Primary Care Psychology services were recruited for the study. Qualitative interview data was transcribed and analysed using NVivo. Thematic Analysis was used to identify themes.

**Results:**

Four major themes were identified: Accessibility, ‘Transitional Object’, Integration, and Trust. Within these 4 major themes, a total of 9 subthemes were identified: Service Accessibility, Immediate Access, Client Engagement, Adjunct-to-therapy, Therapeutic Relationship, Infrastructural Support, Enhancing Treatment, Trust in the Technology, Trust in the Organisation.

**Conclusions:**

Overall, Diffusion of Innovation Theory provides a useful theoretical framework which is consistent with and can adequately account for many of the Major and Subthemes identified in the data. In addition, ‘Transitional Objects’, a key concept within Object Relations Theory, could offer a means of better understanding how patients and professionals engage with digital technology within mental health services particularly.

## Background

### Mobile health and mental health

An increase in demand for mental health services in recent years [1, 2], together with the negative association between service availability and suicide rates found in a number of countries [3], has accelerated interest in the use of mobile health technology to help address access barriers [4]. Digital technology has been found to be beneficial in the delivery of Web-based suicide prevention interventions [5] and public health services are being encouraged to harness such technology to enhance psychological health [6, 7]. In parallel, research in psychiatric outpatient settings, indicate patient desire to use mobile apps to track their mental health [8]. Worldwide ownership of smartphones is in excess of 2.5 billion [9]. In Ireland, smartphone ownership rates rise to 100% for those aged between 18 and 24 years [10]. There are over 10,000 mental health apps available for download across iTunes and Android stores [11]. Case reports and early efficacy studies suggest clinical benefits of mobile health apps in research settings [12, 13] but concerns remain regarding a lack of clinical evidence [14, 15], clinical safety concerns [16], and privacy vulnerabilities [17]. In addition, apps that appear to be effective in research settings are not always equally efficacious in clinical settings [18, 19]. Despite the reported interest from both patients and professionals in utilizing mobile apps [8, 20], poor uptake of such technologies in clinical settings indicates that m-Health resources suffer from low engagement [21].

### Suicide

Suicide is the second leading cause of fatality in 15–29 year olds globally [22]. Suicidal self-directed violence is defined as self-directed behaviour that deliberately casues injury or the potential for injury, where there is evidence of suicide intent [23]. An estimated 25 suicide attempts occur for every death by suicide (100–200 for youth) [24], approximately one-third of those who die by suicide had contact with mental health services in the year preceding death, and one in five had been in contact with a health professional in the month before death [25]. Suicide remains extremely difficult to predict even for very experienced mental health professionals [26, 27]. A recent meta-analysis of 50 years of research highlighted weak progress in our ability to predict suicide and called for a shift in focus from risk *factors* to machine learning-based risk *algorithms* [28]. The lack of consensus in gold-standard suicide risk assessment and management, and the outstanding need for standardized nomenclature, challenge the accurate detection of risk and our ability to prevent suicide outcomes [29]. Data recorded through real-time mobile monitoring may be used to analyse proximal risk mechanisms within the suicidal process and could help to investigate the multi-faceted and inter-dependent relationships put forward by prominent theories of suicide [30, 31].

### Safety planning intervention

The Safety Planning Intervention (SPI) developed by Stanley and Brown [32], was originally designed as a brief intervention for those attending emergency departments at-risk of suicide or following a suicide attempt. The collaborative safety plan, developed by the patient with a trained practitioner and traditionally completed on paper, involves identifying warning signs that indicate the person is approaching a crisis, and developing strategies to enhance that person’s safety. In a cohort comparison study, patients who received SPI with follow-up telephone contact were half as likely to display suicidal behaviour and more than twice as likely to engage with mental health services at 6 month follow-up compared with the control group who received Treatment As Usual [32]. A UK-based study comprising a randomised control trial of a face-to-face safety planning intervention with follow-up telephone contact is currently underway [33]. Recent advancements in mobile technology and increases in its adoption may further improve access to safety planning.

### Engagement

Engagement has posed a challenge for digital health interventions across a broad range of conditions. Activity tracker apps for example, with or without incentives, did not lead to lasting changes in step count [34]. A mobile app designed to be used in the treatment of post-traumatic stress disorder recorded over 166,800 downloads initially reducing to 26,110 users 1 week later [35]. Torous [36] observes that “the degree of engagement is influenced by the depth of the patient’s investment in the interaction with the digital tool; this investment may be defined temporally, affectively, and/or cognitively”. To this end qualitative exploration of user experiences may offer a richer understanding of the barriers and enablers to adopting such technology.

The ability of digital health tools to rapidly iterate offers exponential opportunities to update features but some researchers [37] assert that they also provide a distraction from central issues. Torous and colleagues argue that the field of digital health, as applied to mental health, requires a roadmap, an overarching framework to inform further development, drawing on relevant theories from diverse areas.

### Employee engagement

Engagement is defined as “the measure of an employee’s emotional and intellectual commitment to their organisation and its success” [38]. According to Hewitt and colleagues, characteristics of an engaged employees who keep up to-date with advancement in their field, hold a broader view even at personal cost, are optimistic or positive about their role and their organisation, and work actively to improve things. West and Dawson [39] have highlighted that where employee engagement is high it results in; lower patient mortality, improved clinical care, improved patient experience, improved employee wellbeing, and lower rates of absenteeism. A proposed important factor in attempting to engage employees is the development of trust.

Trust has been defined as “An individual’s expectation that some organised system will act with predictability or goodwill” [40]. In the workplace, it is argued that one of the most distinct advantages of trust is its link to innovation. Workplaces described as ‘high trust’ are reported to find it much easier to embrace organisational change, to adapt faster and achieve better levels of employee engagement. It is also reported to have important benefits for promoting employee well-being and motivation [41].

### Diffusion of innovation

Diffusion of Innovation Theory [42] offers a framework to describe the process through which mental health professionals adopt an innovation. According to this theory, adoption is more likely to occur when the person perceives the idea, product or behaviour, as novel or innovative. The theory identifies a number of stages of adoption which include; awareness of the need for the innovation, the decision to adopt / reject the innovation, the use and testing of the innovation, and continued use of the innovation. The theory proposes that in order for an innovation to be adopted, it must be perceived as offering: a relative advantage, compatibility, complexity, trialability, and observability. The theory may therefore be used in guiding the approaches to be used with different professionals and at different points in the process of innovation adoption. One advantage of this model is that it accounts for how aspects of the technology itself impact on adoption which provides a more dynamic account of the process. One significant limitation of Diffusion of Innovation theory is the comparatively limited attention given to the systems within which innovations are to be adopted.

### Object relations theory

Torous [36, 37], and other researchers have recently discussed the relevance of “transitional objects” and Object Relations Theory [43] more generally to enhancing our understanding of engagement with digital technology within healthcare. From this perspective, the value of the object comes directly from the quality of the relationship, and the object is used as an adjunct to face-to-face contact. Winnicott outlined the importance of what were termed “transitional objects”, a key concept within Object Relations Theory, the commonly used psychological theory of particular relevance in child psychology. In infancy, a child tends to feel unified with their primary caregivers. As the child develops, they begin to display an interest in exploring the world beyond that of their relationship with their primary caregiver. The primary caregiver, from Winicott’s perspective, is unable to meet all of the child’s needs, and the child begins to understand that they must separate from their primary caregiver. The child may find an object, such as a blanket, that helps them cope with the anxiety elicited by separating from their caregiver. From this perspective, the transitional object allows the child to remain connected in a way with their primary caregiver while still being able to independently explore the world.

The current authors have researched the use of mobile health technology in suicide prevention [44, 45], and have engaged stakeholders across mental health services, technology, and suicide prevention policy in the design and development of SafePlan – a Safety Planning app, designed to be used as an adjunct to therapy for individuals aged 16–35 years who are currently accessing mental health services [46]. Members of the research team have also studied the use of Artificial Intelligence and large datasets, in furthering our understanding of suicide risk [47, 48]. Mobile apps, offer a potential means of identifying proximal and distal warning signs of risk which could aid individualised risk assessment and intervention [49]. A pilot Randomised Control Trial of SafePlan [45] in clinical settings is planned. Ahead of this, the researchers engaged mental health professionals in the current study to better understand their experiences of using mobile apps in their practice.

The study aims to address the following research questions:

- What are the experiences of mental health professionals engaged in the use of mobile apps as part of their practise?
- What are the drivers and barriers to the adoption of such technology by mental health professionals?
- What theoretical model best accounts for the experiences of mental health professionals engaged in adopting this technology?

## Methodology

### Research design

This study used a qualitative design given the exploratory nature of the study and need to capture the experience of what is a relatively under-researched group. An Appreciative Inquiry approach [50] was used to engage participants in the generation of a narrative on how mobile health technology could be adopted and integrated into routine mental health care. A fundamental premise of the Appreciative Inquiry approach is that organizations move toward what they study [51] and participants are asked to engage in a dialogue concerning what is needed, in terms of both tasks and resources, to bring about the desired future. Appreciative Inquiry is based on discovery and valuing, envisioning, dialogue and co-constructing the future [52].

According to Sofaer [53] qualitative methods allow people to speak in their own voice, rather than conforming to categories and terms imposed on them by others. Qualitative research has been invaluable in mental health service research, particularly when exploring under-researched areas [54].

Reflexivity within qualitative research invites researchers to explore cause, effect and reflexivity by reflecting on how one views the world [55]. Gianakis and Carey [56] recommend making explicit the reflexivity employed by investigators as a means of identifying and addressing potential biases in the collection and interpretation of data. The first author’s education and training in Clinical Psychology may have biased their knowledge base towards scientific research, theoretically driven hypothesis testing, and traditional methodologies such as randomized controlled trials. These approaches, in the researcher’s view, may fail to capture the complex experience of innovations in health care. In order to better understand the lived experience of mental health professionals who adopt this technology, both an inductive and deductive approach was needed.

### Participants

Fifteen mental health professionals and clinician managers working in the Irish public health service across: Child and Adolescent Mental Health, Adult Mental Health, and Primary Care Psychology services were recruited for the study. A purposive sampling method was utilized, and recruitment occurred through presentation at multidisciplinary team and discipline-specific meetings, internal broadcast emails, and service-wide email invitations to participate. The only inclusion / exclusion criteria were that participants were HSE Mental Health professionals and that they consented to participating.

To enhance generalisability, mental health professionals working in a broad geographical area in the West and Mid-west of Ireland, encompassing different types of mental health services (Primary Care Psychology, Child and Adolescent Mental Health, and Adult Mental Health) and different roles within those services (psychologist, psychiatrist), were included.

The target number of interviewees for this study was 15, the minimum number of interviewees set was 8, and the maximum was 20. Fifteen participants were recruited and completed one semi-structured interview each with the researcher. Interviews were conducted between July and October 2019.

### Materials and procedure

Semi-structured interviews were used to gather qualitative data pertaining to professional’s experiences of using mobile health technology in mental health care. An interview guide was informed by research, theory and practice a priori, with flexibility for the interviewer to explore particular areas of interest or concern to the participant. Sample specific interview guides were used to investigate the experience of enablers and barriers to the integration of digital technology into mental health service provision.

The study employed a data triangulation approach [57, 58]. The multi-site, multi-disciplinary, and multi-service data collection procedures were designed to include a number of different perspectives, across services, and locations. In terms of reflexivity and personal stance, a reflective journal was used to bring researcher biases to conscious awareness.

The interviews lasted between 20 and 60 min. The role of the interviewer was outlined at the beginning of each interview. The interviewee and the interviewer were the only persons present during the interviews. Interview data was collected via dictaphone, transcribed verbatim and stored in line with GDPR standards. The interviewer then checked each transcription against the recording for accuracy and changed all names of individuals and organisations to agreed codes to make them anonymous.

### Data analysis

All data were subject to thematic analysis [59]. Braun and Clarke’s [60] 6-step framework was used: become familiar with the data, generate initial codes, search for themes, review themes, define themes, write-up. Inductive-deductive cycles of thematic analysis were utilized. As the researchers sought to investigate enablers and barriers to the integration of technology, and participants’ experiences of integrating technology into their work, the first step in the analysis took a largely deductive approach. Based on the research questions and a read-through of each transcript, broad codes were identified to be applied to all three data sets. Each data set was coded separately (by the researcher who had carried out the interviews), using the agreed coding framework. In the second analytic step, a largely inductive approach was taken to analysing the coding reports from step one. Data were broken down further to identify specific sub-themes. In the third step, the researchers juxtaposed the resulting sub-themes. The themes and sub-themes are used as headlines in the presentation of results, and quotes from participants are included to illustrate and validate interpretations. To protect anonymity, quotes are identified with participant number only. Unless specifically stated, quotes are selected to represent general themes and opinions in the sample.

### Ethical issues

This study received full ethical approval from the Irish Management Institute Social Research Ethics Committee. Informed consent was sought prior to participation using the study consent form. Participants were informed of their right to withdraw or their right to have their data withdrawn at a later stage.

## Results

### Description of participants

**Fig. 1:**
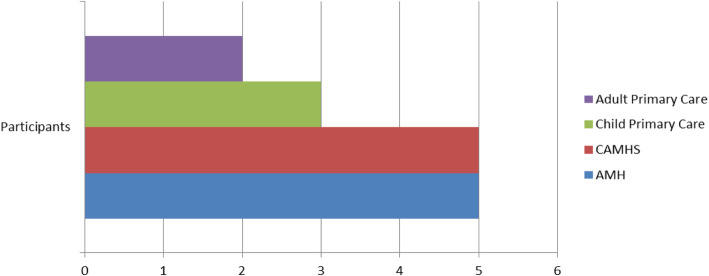
Breakdown of participant professions

Fifteen mental health professionals participated in total: 5 Psychologists working within Adult Mental Health, 3 Psychologists and 2 Psychiatrists working in Child and Adolescent Mental Health, 3 Psychologists from Primary Care Child Psychology Services, and 2 Psychologists from Adult Primary Care Psychology (as outlined in Fig. 1) In terms of geographical spread, Adult Primary Care Psychologists participated from two different sites (one from each), Child Primary Care Psychologists were based at three different sites (one from each), CAMHS Psychologists and Psychiatrists were from two sites (one psychiatrist from each site), five psychologists participated from two different Adult Mental Health sites.

A diverse range of mental health services were represented and participants worked with a broad range of ages and levels of severity of mental health difficulties. Participants were also evenly distributed across Community Healthcare Organisations. Participants were quite experienced, in terms of years spent working in mental health services (Mean 11 years, Range 6–15). The mean age of participants was 39 years, ranging from 35 to 48 years. The majority of participants (*n* = 13) reported that they have experience of recommending a mobile app to services users. A minority (*n* = 3) reported regularly using an app as an adjunct to therapy, no participants reported using an app to support a patient to track their mood or behaviours. However, a number of professionals identified this as a potential advantage of using mobile apps generally.

This sample represents a relatively homogenous professional grouping with the majority of participants being members of the same profession as the researcher (psychology).

#### Thematic analysis

Qualitative data was analysed using thematic analysis.

When discussing the enablers to the integration of technology in mental health services, participants discussed the ‘Accessibility’ offered by the use of technology and described the idea of a mobile app functioning as a ‘Transitional Object’ connecting the patient to their clinician while outside of the clinic setting. When exploring barriers, the challenges of ‘Integration’ into existing infrastructure, and ‘Trust’ both in the technology and in the organisation’s planned use of the technology were discussed by participants.

The four major themes identified were: Accessibility, ‘Transitional Object’, Integration, and Trust. Within these four major themes, a total of nine subthemes were identified: Service Accessibility, Immediate Access, Client Engagement, Adjunct-to-therapy, Therapeutic Relationship, Infrastructural Support, Enhancing Treatment, Trust in the Technology, Trust in the Organisation. An overview of themes is provided in Table 1.

**Table 1:** An overview of Major Themes and Sub-themes

Major Themes	Sub-themes
Accessibility	1. Service Accessibility 2. Client Engagement 3. Immediate Access
Transitional Object	1. Adjunct to Therapy 2. Therapeutic Relationship
Integration	1. Infrastructural Support 2. Enhancing treatment
Trust	1. Trust in the Technology 2. Trust in the Organisation

##### Accessibility

Accessibility was identified as a major theme in the interview data. Participants discussed the extent to which they felt the integration of technology could facilitate greater accessibility to services. This theme comprised of subthemes related to an increase in service accessibility, an increase in engagement with young people in particular who may find traditional mental health service settings inaccessible or stigmatising, and an increase in the accessibility of knowledge and support in times of crisis.

Accessibility was mentioned by the majority of participants specifically (*n* = 9) or related terms were used involving increased capacity to see clients (*n* = 3). Subthemes of: Service Accessibility, Client Engagement, and Immediate Access were identified.

Participants discussed the extent to which digital technology can make services more accessible through direct contact, the provision of information, or as a way of recording or monitoring thoughts, emotional states or behaviour. The potential ability to provide psychological interventions in-situ and when the person may need it were also highlighted.

“Clients could access support whenever they need it and wherever they are” (Participant 11, line 15)

“It would make available an immediate connection that could be flagged in advance and could be used in a crisis”. (Participant 9, lines 34-35).

“I think really it could have the potential to reduce the number of face to face contacts with children and increase the number of children who can access the service” (Participant 15, lines 113-115).

Participants also described the potential for digital technology to help in engaging individuals for whom traditional face-to-face mental health services are not appropriate or accessible.

**“**It would offer another means of engaging service users, particularly service users who find direct social interaction challenging, or who have an interest in technology” (Participant 13, lines 88–90)

In contrast, some participants discussed accessibility as a barrier. In these cases participants were concerned about increased accessibility to smartphones, internet and social media as a result of the mobile app, particularly with children. In this case, the increased accessibility offered was seen as a risk.

“I would be worried I suppose that it would encourage children to have access to a smart phone at an earlier age” (Participant 10, lines 176-177).

##### “Transitional object”

Participants discussed the potential for digital technology to ‘add value’ to face-to-face intervention as an adjunct to therapy. Specifically, participants identified the potential for technology to facilitate the generalisation of skills acquired in clinic to outside of the clinic setting. Additionally, behavioural insights gleaned from recording and monitoring of thoughts, emotions or mood states, were seen as enhancing the work conducted in the clinic.

“Transitional Object” is a commonly used concept within psychology which refers to Object Relations Theory [43]. This theme, encompassing the therapeutic relationship, and the app as an adjunct-to-therapy, was referred to by 8 participants in total in various ways. Many participants pointed to the need for mobile technology to be used as an adjunct to therapy rather than instead of face-to-face therapy.

“Using the phone/app as a transitional object and a means of staying connected with clients outside of sessions” (Participant 11, lines 89–90)

One participant discussed the importance of the Therapeutic Relationship between client and therapist and argued their view of the superiority of face-to-face contact over digitally-based supports. Participants also discussed their concern regarding potential over-reliance on mobile technology in-place of face-to-face interaction.

“There is a need though to prioritise real human face to face interaction within the services and then use technology to support this when that is appropriate”(Participant 9, lines 102-104).

**“**Given that the therapeutic relationship is the strongest agent of change in a therapeutic setting, I wonder about the potential consequences of reliance on technology and screen based interventions. This is not to say I don't support their use, certainly as an add on, but I don't know enough about their use and potential consequences as yet” (Participant 7, lines 92-97).

“With young people we would need to remind them that apps are not a substitute for letting a real person help” (Participant 5, lines 18-20).

The ‘Transitional Object’ theme accounts for all of these views by describing the necessity to generalise coping skills to outside of the therapeutic relationship while also highlighting the primary importance of the therapeutic relationship itself.

##### Integration

Integration was identified as a major theme with participants describing the importance of the app integrating well with existing health service technology, the need for management support and guidance regarding the use of the app, and the need for further training and research to support further integration into services. In addition, participants also discussed the potentially pivotal role an app could play in supporting the integration of skills acquired in therapeutic sessions, to outside of the clinic setting.

“I feel this could help clients to complete between-session work and increase their recording of information. A lot of clients find recording on paper sheets tedious” (Participant 8, lines 81-83).

“It would help clients complete homework tasks and mood and goal monitoring” (Participant 2, lines 15-16).

A number of participants addressed health service infrastructural support for the technology and the extent to which this may impact on the integration of such technology into practise.

“It would need to be available on HSE computers and phones and supported by the HSE’s IT systems” (Participant 7, lines 64-66).

“I think more exploratory research on mobile technology is needed and communication to professionals about the research and the implications for best practice. More practical training on this technology would be helpful” (Participant 9, lines 134-137).

##### Trust

Trust was identified as a major theme and as an overarching concept containing two subthemes. These included (1) Trust in the Technology, and (2) Trust in the Organisation. Three participants discussed the importance of being able to trust the technology (the app itself and supporting infrastructure), and the importance of this in clinical practice.

“usability and reliability of use are so important. Nothing worse than persuading someone to give an app a try and it doesn't function at all or only in a very clunky way” (Participant 14, lines 72-75).

Four participants reported a fear of such technology being used to undermine traditional services if it were to be used by the organisation to save resources.

“My fear would be that it would be used as a cost saving measure only and would undermine the quality of the service” (Participant 15, lines 79-80).

In addition, participants were observant of features in place to protect both clients and professionals.

“If clients record information on their phones and phone gets hacked or stolen there are safeguards in place” (Participant 2, lines 43-45).

## Discussion

The current study aimed to explore the experiences of 15 mental health professionals involved in integrating mobile health technology into their practice. A secondary aim was to identify the drivers and barriers to the adoption of such technology by mental health professionals, and to consider what theoretical models could best account for the data.

Overall, the majority of the mental health professionals who participated in this study identified numerous benefits and potential benefits to adopting mobile technology in their practice. Potential benefits identified included increased accessibility, increased engagement with young people, and the convenience and feasibility of a client using a mobile app in a crisis. Many of the benefits identified are consistent with those asserted by mental health professionals in previous research [20]. A Concern identified by professionals included the potential for the technology to be used instead of, rather than as an adjunct to traditional face-to-face services. Participants noted the importance of the therapeutic relationship in delivering effective interventions and expressed concern about relying on mobile technology in isolation. Such findings are in line with previous research [61], suggesting that although they acknowledge their possible benefits and cost-effectiveness, health care providers are conservative about integrating mHealth technologies in their daily practice. Theoretically, Diffusion of Innovation Theory accounts for many of the themes identified in this research.

### Barriers and enablers

The specific barriers to adoption discussed by participants included trust in the technology (privacy, confidentiality, appropriate and reliable infrastructural support), trust in the organisation (to use the technology as an adjunct-to-therapy rather than instead of traditional services), the need for training, policy guidance, technical support, and a relative lack of research. The barriers identified with regard to trust in technology are consistent with systematic reviews of the app literature calling for further research before such technology is recommended or prescribed [62]. The main enablers identified included the needs addressed by this technology such as to increase accessibility, enhance engagement, enhance the efficacy and generalisation of skills acquired to outside of the clinic setting, provide monitoring and data collection to promote insight. Similar enablers are cited by mental health professionals in previous research [20, 61].

### Theoretical implications

Overall, Diffusion of Innovation Theory [42] provided a useful theoretical framework which accounted for many of the themes discussed by participants. The theory posits that adoption is enhanced when the innovation is seen to address a real need. Rogers defines “relative advantage” as the degree to which an innovation is perceived as better than the idea it supersedes [42]. The five established adopter categories offered a useful way of understanding differing views regarding the potential adoption of this technology in mental health settings.

Specific issues critical to the involvement of professionals within a mental health service in particular, may require theoretical guidance specific to this area. The importance of the therapeutic relationship, and the impact of the quality of this relationship on intervention efficacy and ultimately patient outcomes is well established [63]. The concept of the ‘Transitional Object’ from Object Relations Theory [43] offers a way of understanding how both patients and professionals may engage with mobile technology in mental health services specifically. Viewing the mobile app as a ‘Transitional Object’ places primary importance on the therapeutic relationship with the mobile app working best as an adjunct to traditional face-to-face therapy. This qualitative data therefore supports existing research regarding therapist factors [63]. The terms ‘Transitional Object’ and ‘Object Relations’ are also used routinely by mental health professionals and may promote professional engagement by way of the professional resemblance noted by Rogers [42].

### Integration

The integration of the technology, a major theme here, with existing digital health systems has been raised by researchers previously [64]. In particular, Luxton argues that it is important for behavioral health practitioners to be cognizant of how data is stored and transmitted when integrating smartphones or other mobile technology into their practice. From a legislative perspective, the Health Insurance Portability and Accountability Act (HIPAA) in the US and the General Data Protection Regulation in Europe, are particularly relevant to the sharing of personal health information. Mental health practitioners are also guided by their profession’s code of ethics. For example, the American Psychological Association’s (APA) Ethical Principles of Psychologists and Code of Conduct states that Psychologists should take reasonable precautions to protect confidential information obtained through or stored in *any* medium. Such guidance should be considered therefore when confidential information can be transmitted and stored on smartphones.

### Trust

Some participants discussed trust in relation to their organisation. They were wary that the goal of such innovation was not to improve accessibility but to save resources. Participants also discussed their trust in the technology. Rogers [42] discussed the concept of “trialability” and argues that undertaking a limited cost–benefit trial of an intervention promotes faith or trust that the evidence is correct and that its implementation is logistically possible. Since data collection, the practices of mental health professionals have been necessarily impacted by Covid 19 restrictions [65] and may have offered an opportunity to trial the integration of such technology.

The issue of trust in technology is consistent with existing literature highlighting app safety concerns [16]. Oyungerel and colleagues [66] conducted an analysis of systematic reviews of mobile health applications and found that the overall quality of the evidence of effectiveness was low. The researchers concluded that more robust RCT study evidence is needed, reporting on between-group differences, before mHealth apps could become prescribable. Systematic assessments of apps consistently report serious flaws that may affect service users’ health and wellbeing [67, 68]. There have been calls to improve app oversight, from app libraries and independent expert assessments [69, 70]. Higher standards of app development and quality assurance mechanisms, such as certification or regulation prior to app release to the public have been recommended [71, 72]. The NHS Apps Library [73] or the Psyberguide [69], provide a curated limited collection of apps for users to choose from. Official regulatory bodies such as the FDA and the European CE marking directives have to date only approved a small number of apps [74]. The APA have provided a free mobile app evaluation tool to help psychiatrists select and rate an app [75]. Rodiquez-Villa and Torous [76] propose a dynamic self-certification checklist, validated or challenged by app users, would aim to enhance transparency, engage diverse stakeholders and incentivise the design of safe and secure apps.

### Limitations

While semi-structured interviews provided an opportunity to collect data within a recent area of enquiry which may not be adequately accounted for by available theoretical frameworks, there were considerable limitations to this approach. A significant weakness of this methodology is that it can provide a broad knowledge base which does not lend itself to providing practical action points. The Appreciative Inquiry [50] approach supported this process however, by focusing the interviews on the ways in which innovation could be used to enhance patient care. A limitation of this type of data collection and analysis is the lack of clarity it provides in terms of the impact of a particular strategy or accuracy of a theoretical orientation in bringing about change. However, the chosen process is more dynamic and iterative in nature. Using an Appreciative Inquiry approach allowed the researchers to build on and acknowledge the valued aspects of service provision currently in place. This approach offered a collaborative way of exploring future innovations and how they may be integrated into practice to enhance patient care.

This study endeavoured to gather views representative of mental health professionals within the Irish public health system. It is likely, given the nature of the research and the degree of self-selection involved that the sample included here were predominantly Innovators or Early Adopters and therefore more likely to express positive views towards innovations generally. This theme is consistent with Diffusion of Technology Theory [42], with participants who were already using mobile apps in their practice reporting positive attitudes towards adoption (Innovators), other participants requiring further evidence-based support and information (Early Adopters), and other participants indicating that they would require explicit managerial instruction and policy in order to adopt the technology (Late Adopters). Another limitation of sampling is the lack of professionals outside of psychology and psychiatry who participated. However, many of the themes identified here reflect similar findings in studies of other mental health professional’s experiences of adopting digital technology in their practice [20].

In terms of generalisability to outside of the Irish public health system therefore, the findings, particularly with regards to barriers to adoption, are consistent with research conducted in other countries [20, 61].

## Conclusions and recommendations

Overall, the mental health professional participants in this study reported positive experiences of adopting mobile apps within their practice and identified a number of potential advantages of utilising mobile apps. They identified key drivers to the adoption of mobile apps as the potential for such technology to; increase accessibility to services, enhance engagement, enhance the efficacy and generalisation of skills acquired in session to outside of the clinic setting, and provide monitoring and data collection to promote client insight into their mental health. While some research evidence provides tentative support for the use of mobile apps for the self-management of suicidal ideation [62] consistent with the potential benefits described by participants here, further and more robust research is required to address the efficacy of mobile apps within mental health services. Participants identified trust in the technology (privacy, confidentiality, appropriate and reliable infrastructural support), trust in the organisation, the need for training, policy guidance, and technical support as current barriers to adoption. Many of the barriers identified are consistent with the concerns raised in existing systematic assessments of smartphone tools for suicide prevention [62], and with research findings of professional’s experiences of mobile health technology in other countries [20]. Taken together with previous research [77], the current findings could provide support to mental health professionals in considering the use of mobile health apps as an adjunct-to-therapy in mental health services.

Theoretically, Diffusion of Innovation Theory [42] provides a useful framework which is consistent with and can adequately account for many of the Major and Subthemes identified in the data. In addition, ‘Transitional Objects’, a key concept within Object Relations Theory [43], could offer a means of better understanding how patients and professionals engage with digital technology within mental health services particularly.

The current data, along with broader stakeholder engagement [46], has informed the development of SafePlan (a mobile app-based safety planning intervention designed to be used as an adjunct to therapy), and the implementation of a Pilot Randomized Control Trial of this intervention within Irish public mental health services.

## Data Availability

The datasets used and analysed during the current study alongside study materials (i.e. study consent form) are available from the corresponding author on reasonable request.

## References

[1] McManus S, Bebbington P, Jenkins R, Brugha T (2016). Mental health and wellbeing in England: adult psychiatric morbidity survey 2014.

[2] McManus S, Gunnell D, Cooper C, Bebbington PE, Howard LM, Brugha T, Jenkins R, Hassiotis A, Weich S, Appleby L (2019). Prevalence of non-suicidal self-harm and service contact in England, 2000–2014: repeated cross-sectional surveys of the general population. Lancet Psych.

[3] Tondo L, Albert MJ, Baldessarini RJ (2006). Suicide Rates in Relation to Health Care Access in the United States. J Clin Psychiatry.

[4] Ybarra ML, Eaton WW (2005). Internet-based mental health interventions. Ment Health Serv Res.

[5] Lai MH, Maniam T, Chan LF, Ravindran AV (2014). Caught in the web: a review of web-based suicide prevention. J Med Internet Res.

[6] Assets: how they work. [2019-10-01]. Future in Mind: Promoting, Protecting and Improving Our Children and Young People's Mental Health and Wellbeing. 2015. https://assets.publishing.service.gov.uk/government/uploads/system/uploads/attachment_data/file/414024/Childrens_Mental_Health.pdf.

[7] European Commission. [2019-10-01]. eHealth Action Plan 2012-2020: Innovative Healthcare for the 21st Century. 2012. http://ec.europa.eu/information_society/newsroom/cf/dae/document.cfm?doc_id=4188.

[8] Torous J, Friedman R, Keshavan M (2014). Smartphone ownership and interest in mobile applications to monitor symptoms of mental health conditions. JMIR Mhealth Uhealth..

[9] Taylor K, Silver L. Smartphone ownership is growing rapidly around the world, but not always equally. Pew Res Centre. 2019:4–5.

[10] Deloitte. Global Mobile Consumer Survey 2019: The Irish Cut. Available at: https://www2.deloitte.com/ie/en/pages/technology-media-and-telecommunications/articles/global-mobile-consumer-survey.html.

[11] Torous J, Roberts LW (2017). The ethical use of mobile health technology in clinical psychiatry. J Nerv Ment Dis.

[12] Torous J, Roux S (2017). Patient-driven innovation for mobile mental health technology: case report of symptom tracking in schizophrenia. JMIR Mental Health..

[13] Jonathan GK, Pivaral L, Ben-Zeev D (2017). Augmenting mHealth with human support: notes from community care of people with serious mental illnesses. Psychiatr Rehabil J.

[14] Seppälä J, De Vita I, Jämsä T, Miettunen J, Isohanni M, Rubinstein K, Feldman Y, Grasa E, Corripio I, Berdun J, D'Amico E (2019). Mobile phone and wearable sensor-based mHealth approaches for psychiatric disorders and symptoms: systematic review. JMIR Mental Health.

[15] Bry LJ, Chou T, Miguel E, Comer JS (2018). Consumer smartphone apps marketed for child and adolescent anxiety: a systematic review and content analysis. Behav Ther.

[16] Nicholas J, Larsen ME, Proudfoot J, Christensen H (2015). Mobile apps for bipolar disorder: a systematic review of features and content quality. J Med Internet Res.

[17] Huckvale K, Torous J, Larsen ME (2019). Assessment of the data sharing and privacy practices of smartphone apps for depression and smoking cessation. JAMA Netw Open.

[18] Beard C, Silverman AL, Forgeard M, Wilmer MT, Torous J, Björgvinsson T (2019). Smartphone, social media, and mental health app use in an acute transdiagnostic psychiatric sample. JMIR mHealth uHealth.

[19] Fleming T, Bavin L, Lucassen M, Stasiak K, Hopkins S, Merry S (2018). Beyond the trial: systematic review of real-world uptake and engagement with digital self-help interventions for depression, low mood, or anxiety. J Med Internet Res.

[20] Seibert K, Domhof D, Huter K, Krick T, Rothgang H, Wof-Osterman K (2020). Application of digital technologies in nursing practice: Results of a mixed methods study on nurses’ experiences. ZEFQ.

[21] Ng MM, Firth J, Minen M, Torous J. User engagement in mental health apps: a review of measurement, reporting, and validity. Psychiatr Serv Available at: 10.1176/appi.ps.201800519. 2019. 10.1176/appi.ps.201800519 PMC6839109 30914003

[22] World Health Organization. Suicide prevention. Available at: https://www.who.int/mental_health/prevention/suicide/suicideprevent/en/.

[23] Crosby AE, Ortega L, Melanson C (2011). Self-directed violence surveillance: uniform definitions and recommended data elements, version 1.0.

[24] Maris RW (2002). Suicide. Lancet.

[25] Luoma JB, Martin CE, Pearson JL (2002). Contact with mental health and primary care providers before suicide: a review of the evidence. Am J Psychiatry.

[26] Nock MK, Holmberg EB, Photos VI, Michel BD (2007). Self-injurious thoughts and behaviors interview: development, reliability, and validity in an adolescent sample. Psychol Assess.

[27] Gysin-Maillart A, Schwab S, Soravia L, Megert M, Michel K (2016). A novel brief therapy for patients who attempt suicide: a 24-months follow-up randomized controlled study of the attempted suicide short intervention program (ASSIP). PLoS Med.

[28] Franklin JC, Ribeiro JD, Fox KR, Bentley KH, Kleiman EM, Huang X, Musacchio KM, Jaroszewski AC, Chang BP, Nock MK (2017). Risk factors for suicidal thoughts and behaviors: a meta-analysis of 50 years of research. Psychol Bull.

[29] McLean J, Maxwell M, Platt S, Harris F, Jepson R (2008). Risk and protective factors for suicide and suicidal behaviour: a literature review.

[30] O'Connor RC, Kirtley OJ (2018). The integrated motivational-volitional model of suicidal behaviour. Philos Trans R Soc Lond Ser B Biol Sci.

[31] Joiner TE (2005). Why people die by suicide.

[32] Stanley B, Brown GK, Brenner LA (2018). Comparison of the safety planning intervention with follow-up vs usual care of suicidal patients treated in the emergency department. JAMA Psychiatry.

[33] O'Connor RC, Lundy JM, Stewart C, Smillie S, McClelland H, Syrett S, Gavigan M, McConnachie A, Smith M, Smith DJ, Brown GK, Stanley B, Simpson SA (2019). SAFETEL randomised controlled feasibility trial of a safety planning intervention with follow-up telephone contact to reduce suicidal behaviour: study protocol. BMJ Open.

[34] Finkelstein EA, Haaland BA, Bilger M, Sahasranaman A, Sloan RA, Nang EEK, Evenson KR (2016). Effectiveness of activity trackers with and without incentives to increase physical activity (TRIPPA): a randomised controlled trial. Lancet Diabetes Endocrinol.

[35] Owen JE, Jaworski BK, Kuhn E, Makin-Byrd KN, Ramsey KM, Hoffman JE (2015). mHealth in the wild: using novel data to examine the reach, use, and impact of PTSD coach. JMIR Ment Health.

[36] Torous J, Nicholas J (2018). Larsen ME, et alClinical review of user engagement with mental health smartphone apps: evidence, theory and improvements. Evid Based Ment Health.

[37] Cohen J, Torous J (2019). The potential of object-relations theory for improving engagement with health apps. JAMA..

[38] CIPD (2007). Employee Engagement.

[39] West MA, Dawson JF (2012). Employee engagement and NHS performance.

[40] Maguire S, Phillips N (2008). ‘Citibankers’ at Citigroup: A Study of the Loss of Trust after Merger. J Manage Stud.

[41] Chartered Institute for Personnel and Development (2011). ‘Sustainable organisation performance – what really makes the difference?’ [online].

[42] Rogers EM (1962). Diffusion of innovation.

[43] Winnicott DW (1953). Transitional objects and transitional phenomena; a study of the first not-me possession. Int J Psychoanal.

[44] Melia R, Francis K, Duggan J, Bogue J, O'Sullivan M, Chambers D, Young K (2018). Mobile health technology interventions for suicide prevention: protocol for a systematic review and meta-analysis. JMIR Res Protoc.

[45] Melia R, Francis K, Hickey E, Bogue J, Duggan J, O'Sullivan M, Young K (2020). Mobile Health Technology Interventions for Suicide Prevention: Systematic Review. JMIR Mhealth Uhealth.

[46] O’Grady C, Melia R, Bogue J, O’Sullivan M, Young K, Duggan J (2020). A Mobile health approach for improving outcomes in suicide prevention (SafePlan). J Med Int Res.

[47] Bernert RA, Hilberg AM, Melia R, Kim JP, Shah NH, Abnousi F (2020). Artificial intelligence and suicide prevention: a systematic review of machine learning investigations. Int J Environ Res Public Health.

[48] Barros JM, Melia R, Francis K, Bogue J, O'Sullivan M, Young K, Bernert RA, Rebholz-Schuhmann D, Duggan J (2019). The Validity of Google Trends Search Volumes for Behavioral Forecasting of National Suicide Rates in Ireland. Int J Environ Res Public Health.

[49] Armey M, Schatten H, Haradhvala N, Miller I (2015). Ecological momentary assessment (EMA) of depression-related phenomena. Current Opin Psychol.

[50] Bushe GR, Boje D, Burnes B, Hassard J (2011). Appreciative inquiry: theory and critique. The Routledge companion to organizational change.

[51] Cooperrider DL, Whitney D, Stavros JM. Appreciative inquiry handbook: For leaders of change. 2nd ed. Oakland: Berrett-Koehler Publishers; 2008.

[52] Patkar S, Ashford G. The positive path: using appreciative inquiry in rural Indian communities. Canada: International Institute for Sustainable Development; 2001.

[53] Sofaer S (1999). Qualitative methods: what are they and why use them?. Health Serv Res.

[54] Lyon AR, Ludwig K, Romano E, Koltracht J, Vander Stoep A (2014). McCauley E sing modular psychotherapy in school mental health: provider perspectives on intervention-setting fit. J Clin Child Adolesc Psychol.

[55] Dowling M (2006). Approaches to reflexivity in qualitative research. Nurse Res.

[56] Gianakis M, Carey TA (2011). An interview study investigating experiences of psychological change without psychotherapy. Psychol Psychother.

[57] Denzin N (1978). Sociological methods: a sourcebook.

[58] Patton MQ (2002). Qualitative evaluation and research methods.

[59] Nowell LS, Norris JM, White DE, Moules NJ. Thematic Analysis: Striving to Meet the Trustworthiness Criteria. Intern Jour of Qual Meth. 16(1) 10.1177/1609406917733847.

[60] Braun V, Clarke V (2006). Using thematic analysis in psychology. Qual Res Psychol.

[61] Anastasiadou D, Folkvord F, Serrano-Troncoso E, Lupiañez-Villanueva F (2019). Mobile health adoption in mental health: user experience of a Mobile health app for patients with an eating disorder. JMIR Mhealth Uhealth.

[62] Witt K, Spittal MJ, Carter G, Pirkis J, Hetrick S, Currier D, Robinson J, Milner A (2017). Effectiveness of online and mobile telephone applications (‘apps’) for the self-management of suicidal ideation and self-harm: a systematic review and meta-analysis. BMC Psychiatry.

[63] Strupp HH (2001). Implications of the empirically supported treatment movement for psychoanalysis. Psychoanal Dialogues.

[64] Luxton DD, Bush N, Reger G. mHealth for Mental Health: Integrating Smartphone Technology in Behavioral Healthcare. Profess Psychol Res Pract. 2011. 10.1037/a00024485.

[65] Shore JH, Schneck CD, Mishkind MC. Telepsychiatry and the coronavirus disease 2019 pandemic—current and future outcomes of the rapid virtualization of psychiatric care. JAMA Psychiatry Published online May 11. 2020;(12):1211–2. 10.1001/jamapsychiatry.2020.1643. 10.1001/jamapsychiatry.2020.1643 32391861

[66] Byambasuren O, Sanders S, Beller E, et al. Prescribable mHealth apps identified from an overview of systematic reviews. npj Digit Med. 2018;1(12) 10.1038/s41746-018-0021-9. 10.1038/s41746-018-0021-9 PMC6550270 31304297

[67] Kroenke K, Spitzer RL, Williams JB (2001). The PHQ-9: validity of a brief depression severity measure. J Gen Intern Med.

[68] Lum E, Jiminez G, Huang Z (2019). Decision support and alerts for apps for self-management of blod glucose for type 2 diabetes. JAMA..

[69] PsyberGuide – App review summary. https://psyberguide.org/about-psyberguide/. Accessed 18 Sept 2019.

[70] Torous JB, Chan SR, Gipson SYT (2018). A hierarchical framework for evaluation and informed decision making regarding smartphone apps for clinical care. Psychiatr Serv.

[71] Parker L, Karliychuk T, Gillies D, Mintzes B, Raven M, Grundy Q (2017). A health app developer’s guide to law and policy: a multi-sector policy analysis. BMC Med Inform Decis Mak.

[72] Wykes T, Schueller S (2019). Why reviewing apps is not enough: transparency for trust (T4T) principles of responsible health app marketplaces. J Med Internet Res.

[73] NHS Apps Library. NHS. https://www.nhs.uk/apps-library/?page=1. Accessed 18 Oct 2020.

[74] FDA / CE certified apps directory. Healthskouts. https://apps.healthskouts.com/. Accessed 17 Oct 2019.

[75] American Psychiatric Association. App Advisor; APA App Evaluation Tool. https://www.psychiatry.org/psychiatrists/practice/mental-health-apps. Accessed 01 Nov 2020.

[76] Rodriguez-Villa E, Torous J (2019). Regulating digital health technologies with transparency: the case for dynamic and multi-stakeholder evaluation. BMC Med.

[77] Martinego L, Van Galen L, Lum E, Kowalski M, Subranium M, Car J (2019). Suicide prevention and depression apps’ risk assessment and management: a systematic assessment of adherence to clinical guidelines. BMC Med.

